# Real-Time Sound and Motion Feedback for Violin Bow Technique Learning: A Controlled, Randomized Trial

**DOI:** 10.3389/fpsyg.2021.648479

**Published:** 2021-04-26

**Authors:** Angel David Blanco, Simone Tassani, Rafael Ramirez

**Affiliations:** ^1^Music and Machine Learning Lab, Department of Information and Communications Technologies, Universitat Pompeu Fabra, Barcelona, Spain; ^2^Multiscale and Computational Biomechanics and Mechanobiology Team, Department of Information and Communications Technologies, Universitat Pompeu Fabra, Barcelona, Spain

**Keywords:** music, motor learning, feedback, violin, motion capture, kinematics, e-learning, music learning

## Abstract

The production of good sound generation in the violin is a complex task that requires coordination and spatiotemporal control of bowing gestures. The use of motion-capture technologies to improve performance or reduce injury risks in the area of kinesiology is becoming widespread. The combination of motion accuracy and sound quality feedback has the potential of becoming an important aid in violin learning. In this study, we evaluate motion-capture and sound-quality analysis technologies developed inside the context of the TELMI, a technology-enhanced music learning project. We analyzed the sound and bow motion of 50 participants with no prior violin experience while learning to produce a stable sound in the violin. Participants were divided into two groups: the experimental group (*N* = 24) received real-time visual feedback both on kinematics and sound quality, while participants in the control group (*N* = 26) practiced without any type of external help. An additional third group of violin experts performed the same task for comparative purposes (*N* = 15). After the practice session, all groups were evaluated in a transfer phase without feedback. At the practice phase, the experimental group improved their bowing kinematics in comparison to the control group, but this was at the expense of impairing the sound quality of their performance. At the retention phase, the experimental group showed better results in sound quality, especially concerning control of sound dynamics. Besides, we found that the expert group improved the stability of their sound while using the technology. All in all, these results emphasize the importance of feedback technologies in learning complex tasks, such as musical instrument learning.

## 1. Introduction

### 1.1. Technology-Enhanced Music Learning

Audio-based and motion capture technologies could provide us with objective measures of student improvement in musical instrument performance. This could allow music teachers to monitor their students' learning process to provide better and personalized learning strategies. This is even more important when we take into account that traditional teaching methods of musical performance movement may not be based on the understanding of its biomechanics components but on the subjective and vague perception of human movement (Brandfonbrener, 2004). Moreover, learning to play an instrument is based on a master-apprentice relationship which consists of weekly lessons, followed by long periods of self-study. According to Welch (1985), this could dissociate the teacher's feedback from student's online proprioceptive and auditory sensations that follow his/her performance.

Using the violin as a case of study, the TELMI Project (Technology Enhanced Learning of Musical Instrument Performance)^1^ had the general objectives to design and implement new technologies for music learning and training (based on multi-modal-feedback-technologies, such as audio, image, video, and motion), together with the evaluation of their pedagogical effectiveness. Together with other bowed-string instruments, the violin requires special considerations compared with other instruments. The process of good sound generation in the violin is a complex task that requires coordination and spatiotemporal control of bowing gestures (Schoonderwaldt and Demoucron, 2009). More than 700 practice hours are needed to achieve bowing skills comparable to those of experts according to Konczak and Jaeger (Konczak and Jaeger, 2009). Because pitch control in the violin is continuous, playing with correct intonation becomes a central issue of violin performance (just as it happens with the voice). And, finally, string players also have the highest risks of playing-related musculoskeletal disorders with the shoulder and the neck being the main body parts affected (Fishbein et al., 1988).

In this work, we aim to evaluate some of the technologies developed inside the context of the TELMI project. In particular, we study, through a controlled and randomized experimental design, the effects of real-time augmented feedback in learning bow control within a group of 57 participants with no prior experience playing the violin or any other bow-string instrument. The technologies evaluated in this experiment are capable of offering augmented feedback on bow kinematics (Vamvakousis et al., 2018) as well as on sound quality (Giraldo et al., 2018). The features that are usually considered as important for beginners to take into account when learning when learning to control the bow are related to the kinematics of the up and down movements, as well as with the force and speed exerted on the strings. All those aspects can disrupt the quality of the desired sound coming from the instrument, being the reason why the presence of this type of feedback could also be of great benefit.

### 1.2. Background

#### 1.2.1. Good Posture and Bowing Technique

Some initial tools based on gesture analysis can be found in the i-Maestro project (Ng and Nesi, 2008). Since then, different techniques have been used to study posture and bowing techniques for the violin. For example, Schoonderwaldt and Demoucron (2009) extracted bowing parameters from violin expert performance by combining optical motion capture with sensors (see also Schoonderwaldt and Wanderley, 2007; Deutsch, 2011). Low-cost methods have also been investigated to track violin performance gestures. For example, through indirect-acquisition-techniques using audio information (Perez Carrillo and Wanderley, 2012), by using resistive fingerboard and optical reflectance sensors placed on the bow stick (Pardue et al., 2015), or, more recently, by the use of an infrared depth camera (Vamvakousis et al., 2018).

However, little has been done to explore the educational potential of these technologies yet. As Visentin et al. (2008) remarked, the similarities of violin performance with other already tested paradigms in the area of kinesiology are important (Hay, 1993). This means that some of the methodologies which are successful in those areas (including the use of tracking systems to evaluate the effects of training) may have the potential to be used to maximize performance or reduce the risk of injury in violin performance. For that purpose, the finding of common patterns of expert performance employing tracking technologies is an essential part of assessing the learning progress in novice players. Recent studies have been done in that direction (Peiper et al., 2003; Visentin et al., 2008; Konczak and Jaeger, 2009; Verrel et al., 2013; Dalmazzo and Ramírez, 2019; Volta and Volpe, 2019).

One of the first skills a novice violin student has to learn is “straight bowing.” A common mistake by beginner-violin-students is not to keep the bow parallel to the bridge and perpendicular to the strings. “Round bowing,” as it is called, is said to obstruct the quality of the sound as it makes it difficult to control the contact point between the bow and the string. This contact determines the distance between the bow and the bridge, which directly affects sound production. Van Der Linden et al. (2011) presented and evaluated a system specifically designed for that purpose called MusicJacket. MusicJacket is a wearable system that tracks a player's bowing action and provides vibrotactile feedback whenever the player deviates from a target trajectory. After six training sessions, the authors found a general improvement trend in the test group throughout sessions, although no significant results were found in comparison with the control group at the retention test where the technology was absent. The employed sample of participants and the difficulty of the task was probably an important limitation (four per group). Another important limitation is that “straight bowing,” despite being an essential factor for obtaining a good sound, can hardly be considered by itself an indicator of sound improvement on the violin. Taking into account that both sound and gesture are important features to be considered together, new efforts are being made in the direction of finding audio features to characterize sound quality.

#### 1.2.2. Sound Quality Detection

Probably, some of the first attempts to identify descriptors that could be correlated with the quality of the sound can be found in the work of Romaní et al. (2015). Romaní et al. correlated the subjective opinions about sound quality of professional musicians, after listening to single notes recordings of their own instrument, with audio features extracted from the recordings. Those features were extracted using Essentia (Bogdanov et al., 2013). Based on their work, an educational app called Cortosia (Korg, 2018) was implemented to offer visual feedback to music students about the quality of their produced sound. Posteriorly, Giraldo et al. (2018) implemented a real-time feedback system of sound quality by using machine learning models based on different tone examples recorded by a professional violinist.

By using some of the previous audio descriptors, such as *dynamic stability* or *pitch stability*, in a previous study we implemented an offline sound quality visual feedback system (SQVFS) (Blanco and Ramirez, 2019). It was evaluated in an experiment with both expert violinists and non-violinists. The use of an expert group allowed us to posteriorly replicate the validity of those descriptors to differentiate between beginners and experts. The descriptors also demonstrated their value as a reference for tracking the improvement of the participants throughout the session. Furthermore, receiving feedback from the SQVFS allowed the test group to stay engaged and improve their scores at the end of the session compared with the control group who stabilized results after the first block of trials.

#### 1.2.3. Recent Views on Motor Learning

Motivational and social factors are known to influence learning but also motor learning in general (Locke, 1966; Wulf and Lewthwaite, 2016). Regarding music learning, Demorest and Pfordresher (2015) stated that it can be difficult for music students to develop their singing abilities if singing was viewed as a fixed characteristic (like a “talent”) rather than a temporary condition that could be improved. Even more, it is well-known from a large list of studies in motor learning (and learning in general) that making efforts in changing this kind of conceptions of ability (as a fixed capacity vs. being amenable to change with practice) can enhance motor learning (Dweck and Leggett, 1988; Jourden et al., 1991; Mangels et al., 2006; Blackwell et al., 2007; Wulf and Lewthwaite, 2009). According to Wulf and Lewthwaite (2016), this is possible due to the enhancement of expectancies which can influence working memory, long-term memory, and attentional capture (Zanto et al., 2010; Shomstein and Johnson, 2013; Jiao et al., 2015).

### 1.3. Aims of the Study

In this study, we aimed to evaluate in an experimental setup different modalities of SkyNote, a novel tool designed to offer feedback in real-time to violin players. We designed an experiment with both professional violinists and beginners with little or no musical experience to evaluate both the effects of real-time visual motion capture feedback on “straight bowing” (as Van Der Linden et al., 2011) combined with the effects of real-time sound quality feedback. We expected that the evaluation of both indicators would offer us a wide picture of the effects and the impact these technologies can have on learning. Participant's skills were first evaluated in a *Baseline* condition which was followed by an *Acquisition* condition where one group of participants received real-time feedback from SkyNote while a control group just received oral instructions. Finally, participants took part in a *Transfer* condition to study the retention effects.

In general terms, in this study we seek to answer the following questions:

1. Does real-time visual feedback improve the bowing technique and sound stability in violin beginner students?
2. Is this improvement retained after removing the real-time feedback?

We decided to include an expert group in the analysis. If some of the computed descriptors allow us to differentiate between beginners and experts we will consider them potential descriptors of violin performance. What is more, if throughout the session the beginner's results of those potential descriptors resemble those of an expert, we will consider that the participant has *improved* his/her results in those specific variables. As already shown in previous research (Romaní et al., 2015; Blanco and Ramirez, 2019), we expected that variables, such as *dynamic stability* or *pitch stability* would be potential descriptors of the quality of the generated sound. We also expected descriptors, such as *bow skewness* (i.e., how straight is the bow during the performance) could be a potential descriptor of violin performance as has already been used in previous studies (Van Der Linden et al., 2011).

We decided to deliver in different conditions the feedback related to sound quality from the feedback related to bow kinematics. That is, participants from the feedback group took part in two different conditions, each one biasing the focus of their attention on a particular modality by offering sound feedback or motion feedback. Participants from the control group also participated in two different conditions, but instead of receiving feedback, they were explicitly asked to focus their attention on a particular modality when performing the required exercise. Previous studies which evaluated the effects of real-time feedback have shown that although a pattern of worsening results appeared at the moment of receiving feedback, it was compensated with higher improvements at the Transfer conditions (Welch et al., 1989; Wilson et al., 2005; Paney and Tharp, 2019). The reasons attributed to these events are usually related to an increase in cognitive load at the time of receiving the feedback. We expected to find a similar trend with our participants.

We asked participants at the end of the experiment to fill a questionnaire with questions regarding their satisfaction with the technology together with which were the most common problems they faced when using it.

## 2. Materials and Methods

### 2.1. Participants

Fifty-seven participants with no prior violin playing experience were recruited from the university campus to participate in an experiment in which they were told they would receive a free violin lesson. In addition, 15 expert violinists with at least 7 years of experience [EG; eight women, seven men; mean age: 32.4 (10.06); mean years experience: 18.6 (5.53)] were recruited from both the university campus and different music schools and conservatories in Barcelona. Participants provided their written consent and procedures were approved by the Conservatoires UK Research Ethics committee on 04/04/2017, following the guidelines of the British Psychological Society. Participants also filled a questionnaire about their musical skills, main instrument, and years of music training. Beginner participants were randomly split into two different experimental groups: the Feedback Group [FG; 15 female, 14 male; mean age: 29.915 (4.88)] and the Control Group [CG; 19 female, nine male; mean age: 28.91 (7.5)]. All participants reported having received 1 year or less of formal training in a musical instrument [mean: 0.06 (0.23) years]. The study was carried out in one recording studio located in the Information and Communication Technologies Engineering (ETIC) department of the Universitat Pompeu Fabra, Barcelona.

### 2.2. Experimental Procedure

#### 2.2.1. FG and CG

Before starting the experiment both groups of beginners took part in a practice session. In that practice session, they were instructed on violin technique, bow position, stance, and bow grip through the Youtube video which explained some of the most important concepts to realize the required full bow exercises correctly (see section 2.3 for more details). A full bow exercise consisted of the alternation of two up and down bowing movements using the full length of the bow with the goal of producing a stable and clear sound. Participants could play while watching the video and explore creating sound with the violin, they could also rewatch different parts of the video while practicing. Participants were informed about the main variables we will use to evaluate their performance: *bow skewness* (bowing parallel to the bridge), *contact point* (measured as bow-bridge distance), *inclination* (taking care of not playing the other strings during the movement with the bow), *pitch stability* (related to avoiding scratchy sounds), and *dynamic stability* (trying to maintain the energy of the sound stable during the whole exercise, even during up-to-down or down-to-up changes). They also were encouraged to explore how pitch changes when they displace their finger down the fingerboard (the sound produced has a higher pitch) or when they displace it further away (produces a lower pitch). The duration of this practice session was around 16 min (6 min video + 10 min practice).

The experiment consisted of three blocks: *Baseline* (10 trials), *Acquisition* (35 trials), and *Transfer* (10 trials). In each trial, participants had to locate in the fingerboard of the D string the location of the five different musical target notes that were displayed triggering the reference synthetic sound (RSS) of the system, which was a pure tone at the chosen frequency. Then, while centered in front of the Kinect camera, they were asked to perform a full bow exercise taking into account what they learned in the practice session and while the system recorded their sound and motion descriptors (see section 2.4 for more details about the system used). We also collected their pitch deviation from the RSS. However, results related to intonation and pitch matching skills will be reported in an accompanying paper.

**Figure 1:**
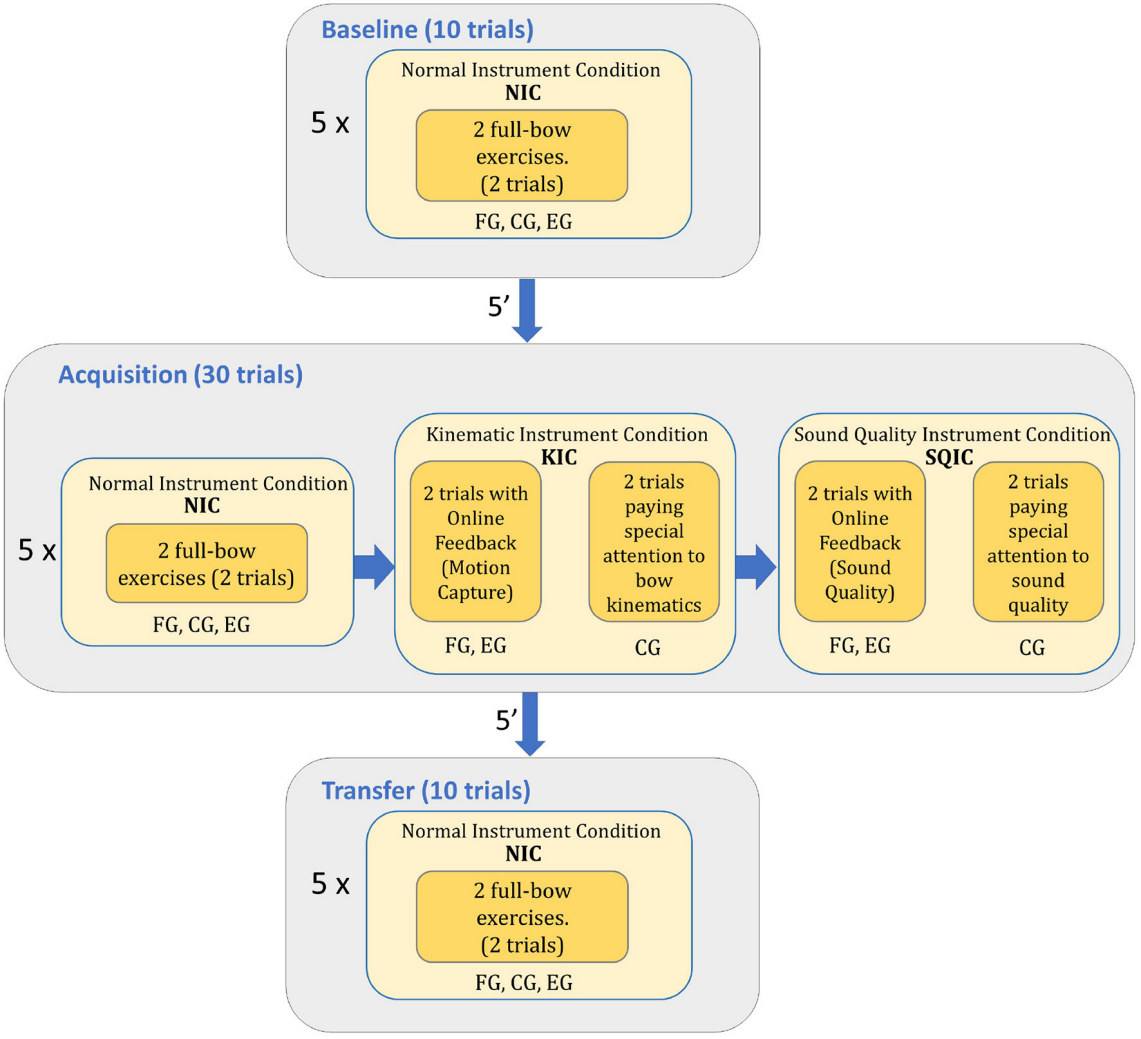
Diagram with the different blocks of the experiment and the different conditions each group of participants went through.

Both the *Baseline* and *Transfer* blocks were equal for all the participants. They consisted of five sub-blocks of two trials each (10 trials in total) where participants had to perform a full bow exercise in each one of them. The Acquisition block however differed between the groups although the total number of trials remained the same. It consisted of five sub-blocks with six trials per block that were performed under different conditions. The first two trials of each sub-block were performed under the *Normal Instrument Condition* (NIC) that consisted of two normal full bow exercises as those performed in the *Baseline* and *Transfer* blocks. The third and fourth trials were performed in a row in the *Kinematic Instrument Condition* (KIC) and it was different for each group of beginners. While performing the full bow exercises, the FG received real-time visual feedback (RTVF) on kinematics allowing them to correct their bow movements when they were not parallel to the bridge (i.e., improving *bow skewness*) or maintaining stable other important variables, such as *bow-bridge distance* or *inclination*. On the other hand, the CG was asked to perform full bow exercises as usual but placing special attention to the demanded kinematic variables and not paying so much attention to the produced sound. Finally, the fifth and last trial of the sub-block was called the *Sound Quality Instrument Condition* (SQIC) and it was also different for each group of beginners. The FG received RTVF on sound quality while performing two more full bow exercises allowing them to see in real-time the score of the descriptors *pitch stability* and *dynamic stability*. On the other hand, the CG was asked again, to perform the full bow exercises in a row and to pay attention to the quality of their sound and to the demanded sound quality variables. Between the NIC and the KIC there was a condition called the *Pitch Instrument Condition* (PIC). In that condition participants had the option to correct their previous decision regarding pitch after receiving different types of augmented feedback. Based on the assumption that pitch-matching skills should not interfere with bowing technique in the violin, details regarding the different types of feedback studied to improve intonation will be discussed in an accompanying article. In Figure 1 we can see a summary of the different blocks of the experiment.

After the *Baseline* block and before the *Acquisition* block participants rewatched the instructional video and remained about the main variables that will be used to evaluate their performance. In addition, the real-time feedback was presented to the FG who received special instruction for its interpretation. On average, between one block and the other, participants rested around 5 min. The duration of the experiment tended to last between 1 and 1 h and a half. At the end of the *Transfer* block, those groups of participants who did not receive RTVF from the software (i.e., the CG) could experiment and practice freely with SkyNote and explore each one of the different feedback modalities the rest of the groups used (pitch, kinematic, and sound quality). After the experiment, all groups of participants answered a questionnaire giving their opinion regarding the technology seen.

#### 2.2.2. EG

Before starting the experiment the EG watched the last part of the instructional video which contained a visual example of how to perform the exercise to make sure they understood the task. They were also informed about the main variables that would be used to evaluate their performance. Like those in the FG, the EG also received the same feedback in both KIC and SQIC. Finally, the EG also answered the same questionnaire giving their opinion regarding the technology seen.

### 2.3. Learning Materials

Basic information about violin playing techniques like stance, violin position, bow position, and grip was delivered to the beginner participants through one didactic Youtube video of a professional violinist before the experiment^2^. The video covers some aspects, such as *contact point*. The *contact point* is the point on the string where the bow force is applied, and needs to be located between the bridge and the fingerboard for good sound results. Thus, participants should maintain a constant contact point during the exercise. The video also covers the relation between speed and force, i.e., if you displace more force on the string you should move the bow faster to avoid “scratchy” sounds in the violin, otherwise if you displace less force you should move the bow slower to avoid “whistling” sounds). At the end of the explanation, there is a visual example of how to perform full bow exercises (alternation of up and down movements using the full length of the bow) focusing attention on bowing parallel to the bridge and how to move the wrist of the right hand to achieve a straight bow movement. The duration of the video is about 6 min. The EG visualized only the last part of the explanation to make sure they understood the task.

### 2.4. Providing Visual Feedback With SkyNote

The system we used to deliver real-time feedback to participants, SkyNote, is one of the main outcomes of the TELMI Project (Ramirez et al., 2018). SkyNote is an integrated system that combines different technologies for real-time feedback on pitch, intonation, dynamics (Mayor et al., 2009), motion capture (Vamvakousis et al., 2018), and tone quality (Giraldo et al., 2018). This feedback can be displayed in customized widgets or directly on the musical score, allowing for real-time experimentation and overall performance evaluation. However, for this experiment, we presented feedback of a single performance aspect at a time.

**Figure 2:**
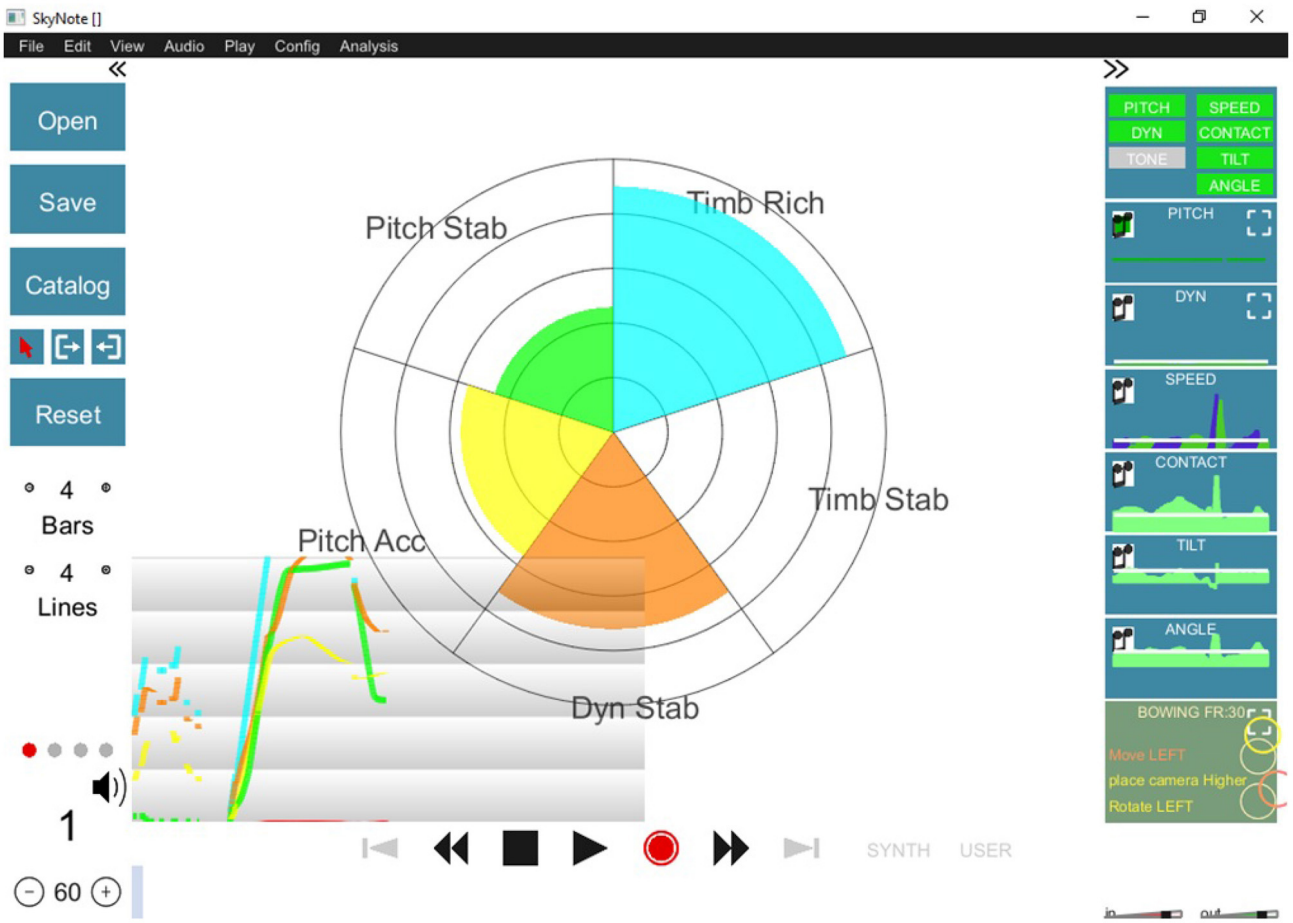
Visual display of the tool used to offer real-time sound quality feedback to participants. Each portion of the spider chart represents a different sound feature while its amplitude represents how close the participant was to the ideal sound.

Figure 2 shows the display used for the real-time feedback used for tone quality. Several descriptors, such as “Pitch Stability” and “Dynamic Stability” appear represented in a spider chart delivering online feedback about the score of each one of the descriptors used (for more details see Giraldo et al., 2018).

**Figure 3:**
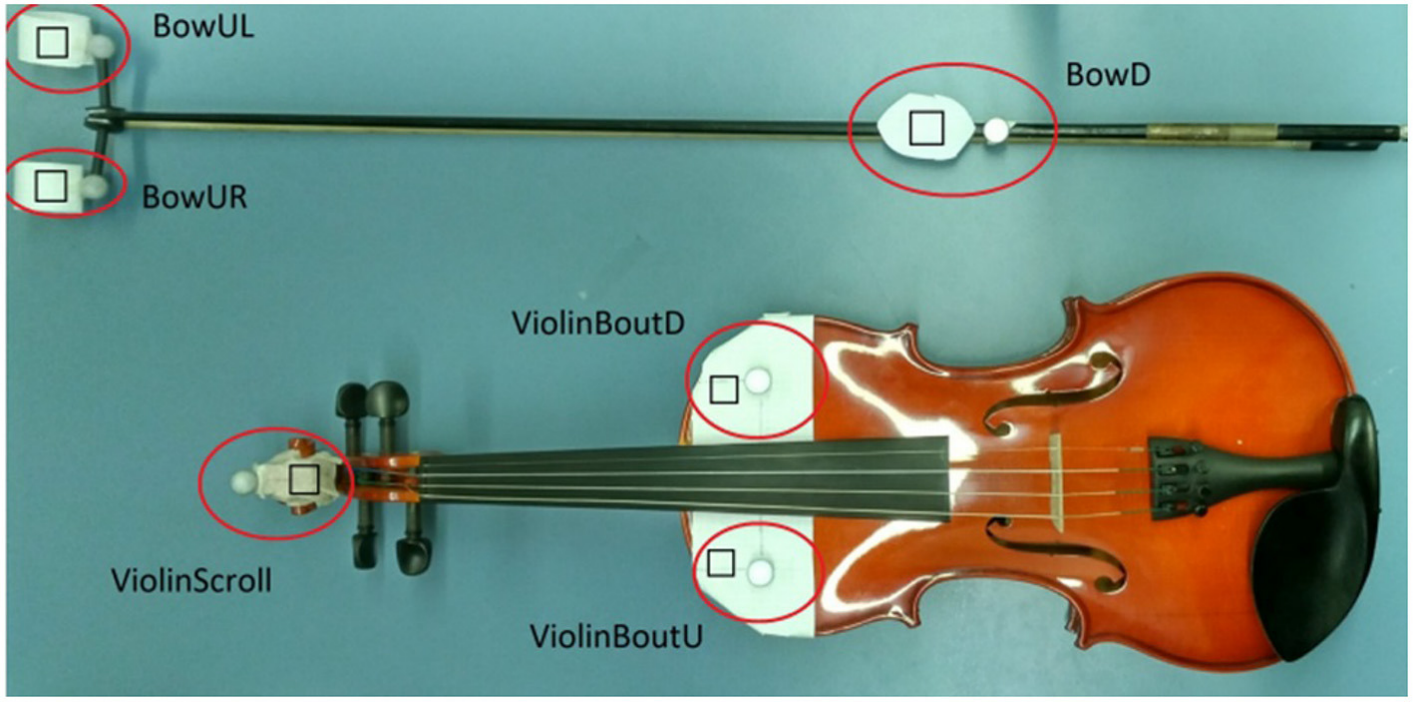
These markers, when placed in the bow and the violin, allow SkyNote to track the bow movement of the participant.

**Figure 4:**
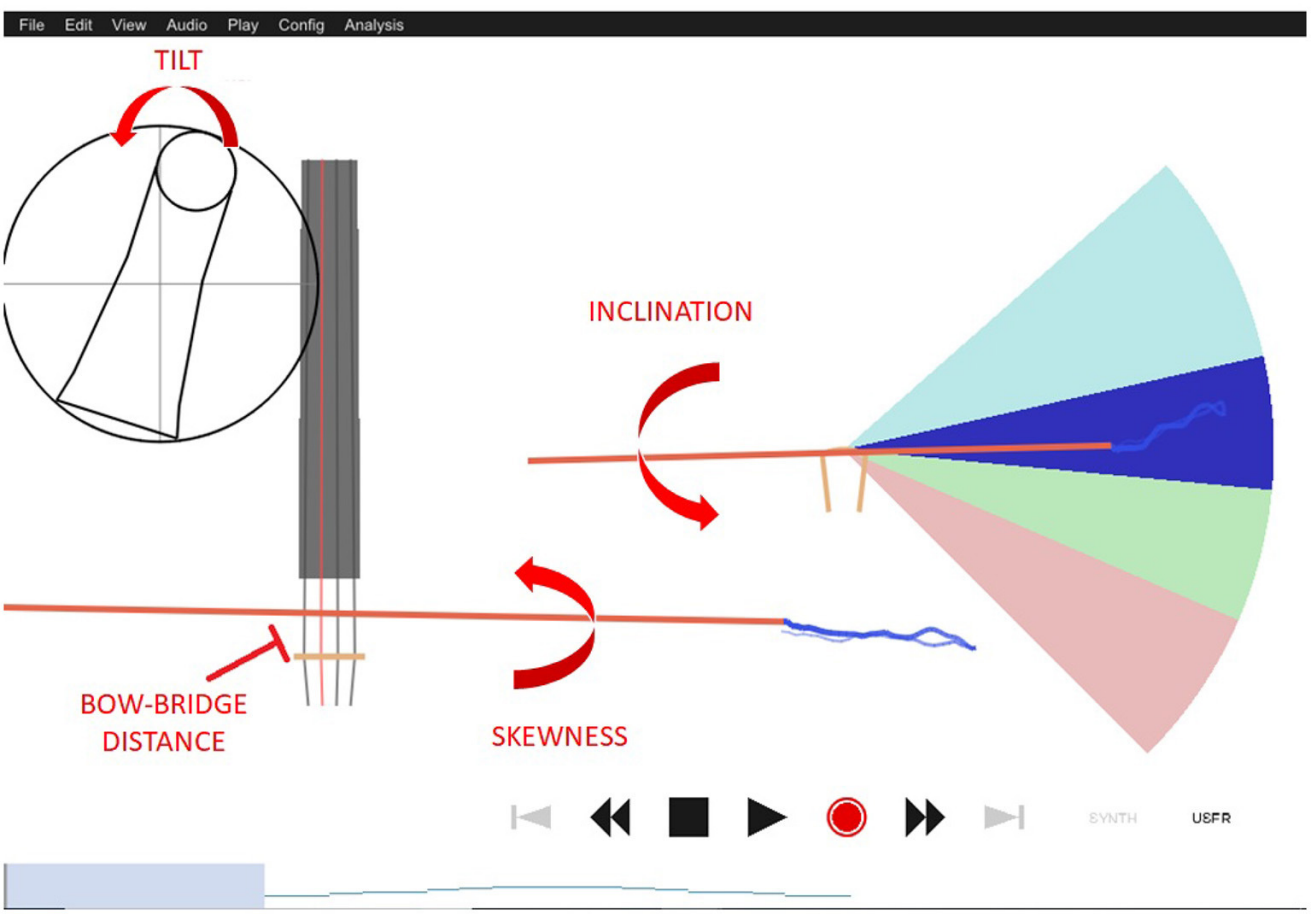
Visual display of the tool used to offer real-time feedback on motion and kinematics to participants. From the display, we can infer some of the descriptors that will be computed later on. The value of skewness, for example, is close to 0 when the bow remains perpendicular to the strings as is the case in this figure.

The system can also monitor specific aspects of the bowing technique when a motion-tracking device is attached (i.e., a Microsoft Kinect) and some markers are placed on the bow and the violin (see Figure 3). Some of these aspects include bow tilt, speed, weight, contact point, inclination, and direction. In Figure 4 you can see the online display on kinematics used for the experiment. For more details see Vamvakousis et al. (2018).

We used an omnidirectional condenser microphone (Behringer, 2013) mounted on a stand to record the audio during the session. One NUC computer to run SkyNote, and two screens: one to deliver feedback to the participant and the other one for the experimenter. The feedback screen could be locked or unlocked by the experimenter based on the condition or group to which the participant belongs.

### 2.5. Questionnaires

Using a questionnaire developed inside the context of the TELMI project we collected some of the views of the participants after the experiment in 27 questions. The questionnaire is available online^3^. Questions related to the usability of the technology were ignored as, in this experiment, participants did not operate the tool (but the experimenter). The questionnaire could be separated into four different sections of questions: questions related to satisfaction with the technology, perception of their own performance, effectiveness of the augmented feedback delivered and problems found with augmented feedback.

The *Satisfaction* questions of the questionnaire had the following form:

- To what degree this tool (from 1 *not satisfied at all*, to 5 *very satisfied*)
  □ #2…help you learn more quickly?
  □ #3…improve your performance?
  □ #4…increase your productivity?
  □ #5…increase the effectiveness of your practice?
  □ #6…make practicing easier?
  □ #7…useful?

- How likely are you to (from 1 *not at all likely*, to 5 *very likely*)
  □ #25…continue using this tool
  □ #26…recommend this tool to others

Questions #16 and #17 were related to the effectiveness of each one of the technologies used.

- Rate the Technology (from 1 *not effective*, to 5 *very effective*)
  □ #16…Timber Stability
  □ #17…Kinect and motion detection

Question #18 was related to the *perception of their own performance*.

- #18 What do you think has improved more during the session? Select one answer:
  □ Pitch-Matching
  □ Timber
  □ Motion and Kinematics
  □ Others.

Questions 20 to 24 were related to the problems found with the augmented feedback. Questions were presented in the form of statements. Answers were from 1 *Strongly Disagree* to 5 *Strongly Agree*:

- #20 Feedback too fast to follow
- #21 Too much feedback information
- #22 Feedback difficult to understand
- #23 Cannot play while watching the feedback.

## 3. Sound and Motion Analysis

All the data was processed in Matlab (MATLAB, 2010), analyzed in Weka (Frank et al., 2016), and in SPSS (IBM Corp, 2011). All the raw data, wav files and statistics for each participant are freely available from Zenodo (Blanco et al., 2020, 2021).

### 3.1. Kinematic Features

Figure 4 shows an example of some of the parameters extracted from the exercise of each participant.

- Position: Refers to the distance between the contact point to the frog computed as the euclidean distance.
- Velocity: The derivative of bow position.
- Bow-bridge distance: Distance between the contact point and the bridge.
- Inclination: The first euler angle (roll) of the bow rigid object in the violin coordinate system.
- Tilt: The second euler (pitch) angle of the bow rigid object in the violin coordinate system.
- Skewness: The third euler angle (yaw) of the bow rigid object in the violin coordinate system.
- Bow-violin distance: the distance between the bow and the violin itself.

Each feature was extracted with a sampling rate of 86.13 samples/s. The skewness angle, as defined here, has a value of 0 when the bow is completely perpendicular to the strings. For each trial, we computed the *bow skewness* descriptor as the mean absolute error of the skewness angle referenced to zero (see Equation 1).

(1) $$\begin{array}{r} bowSkewness=\frac{1}{N}\sum_{i=1}^{N}\left|angle-0\right| \end{array}$$

Where *angle* is the third euler angle measured and 0 the reference. N is the number of samples in a trial.

### 3.2. Sound Quality Features

Sound quality features were extracted in the same manner as in Blanco and Ramirez (2019). We used the Yin algorithm (Llimona, 2015) to extract the fundamental frequency (f0), instantaneous power, and aperiodicity from the audio signal of each trial using a window size of 33 ms with a hop size of 0.7 ms. The quality of the sound recorded in one trial was assessed through sound descriptors, such as *dynamic stability* (Equation 2) or *pitch stability* (Equation 3) by computing the standard deviation of both f0 and power, respectively throughout the trial (Romaní et al., 2015). Equations (2) and (3) provide a formal description of these descriptors.

(2) $$\begin{array}{r} dynamicStability=\sqrt{\frac{1}{N}\overset{N}{\underset{i=1}{\sum }}{\left(P_{i}-\mu \right)}^{2}} \end{array}$$

(3) $$\begin{array}{r} pitchStability=\sqrt{\frac{1}{N}\overset{N}{\underset{i=1}{\sum }}{\left(f0_{i}-\mu \right)}^{2}} \end{array}$$

Where N is the number of samples in a trial. *Pi* is instantaneous power in Db. *f0i* is the instantaneous fundamental frequency in Hz and *μ* is the mean value of, respectively, the power (Equation 2) or the fundamental frequency (Equation 3) calculated over the trial. Note that in this definition of the descriptors lower values indicate *more* stability while higher values indicate *less* stability.

### 3.3. Statistical Analysis

We performed two different analyses of the data using SPSS. One for the kinematic results and another one for the sound quality results.

Because we wanted to evaluate the importance of some of the kinematic descriptors extracted to differentiate between beginners and experts, we performed a 3 × 5 mixed-design for each analysis with Group (FG, CG, and EG) as between-subject factors and Condition (*Baseline, Acquisition-NIC, Acquisition-KIC, Acquisition-SQIC*, and *Transfer*) as the within-subject factor. For the kinematic analysis, the mixed-design was univariate with the results of *bow skewness* for each condition while for the sound quality analysis it was multivariate with the results of *dynamic stability* and *pitch stability* for each condition. *Post-hoc* tests using the Tukey method for multiple comparisons with Bonferroni correction were performed between the groups of participants. The descriptors that showed significant differences between the experts and both groups of beginners were considered as good evaluators of performance.

To study the impact of real-time feedback in our beginner's groups we needed to look for possible interactions between both beginner's groups and conditions for those variables previously considered. For that purpose, we performed a 2 × 5 mixed-design with Group (this time only FG and CG) as between-subject factor and Condition as the within-subject factor. For the descriptors that showed a significant interaction between Condition and Group, a posterior simple main effect analysis was performed on each group to find out which conditions were causing the interaction. Pairwise comparisons tests were performed between the conditions using the Bonferroni correction.

Finally, to compare the effect of training with SkyNote in the amount of improvement, we performed three independent *t*-tests of the relative difference between the *Transfer* and the *Baseline* for each one of the descriptors applying Bonferroni correction.

Before running the analysis discarded all the participants who declared to be left-handed (two from the CG and four from the FG) together with one participant from the FG who declared having already received violin lessons as a child. Given that we found deviations due to bad Kinect camera tracking not related to the actual performance of participants (and thus other modalities were not affected), we decided to separately perform the outlier analysis for each modality. We also removed four participants from the CG and three more from the FG in the kinematic analysis because they were labeled as outliers (values bigger than three interquartile ranges). Finally, we removed one participant from the CG and one from the EG in the sound quality analysis for the same reason. After removing the outliers all the data passed the assumptions of normality required to perform the tests. All the results presented in the following sections were Greenhouse-Geisser corrected.

## 4. Results

### 4.1. Analysis of Differences Between Experts and Beginners

**Figure 5:**
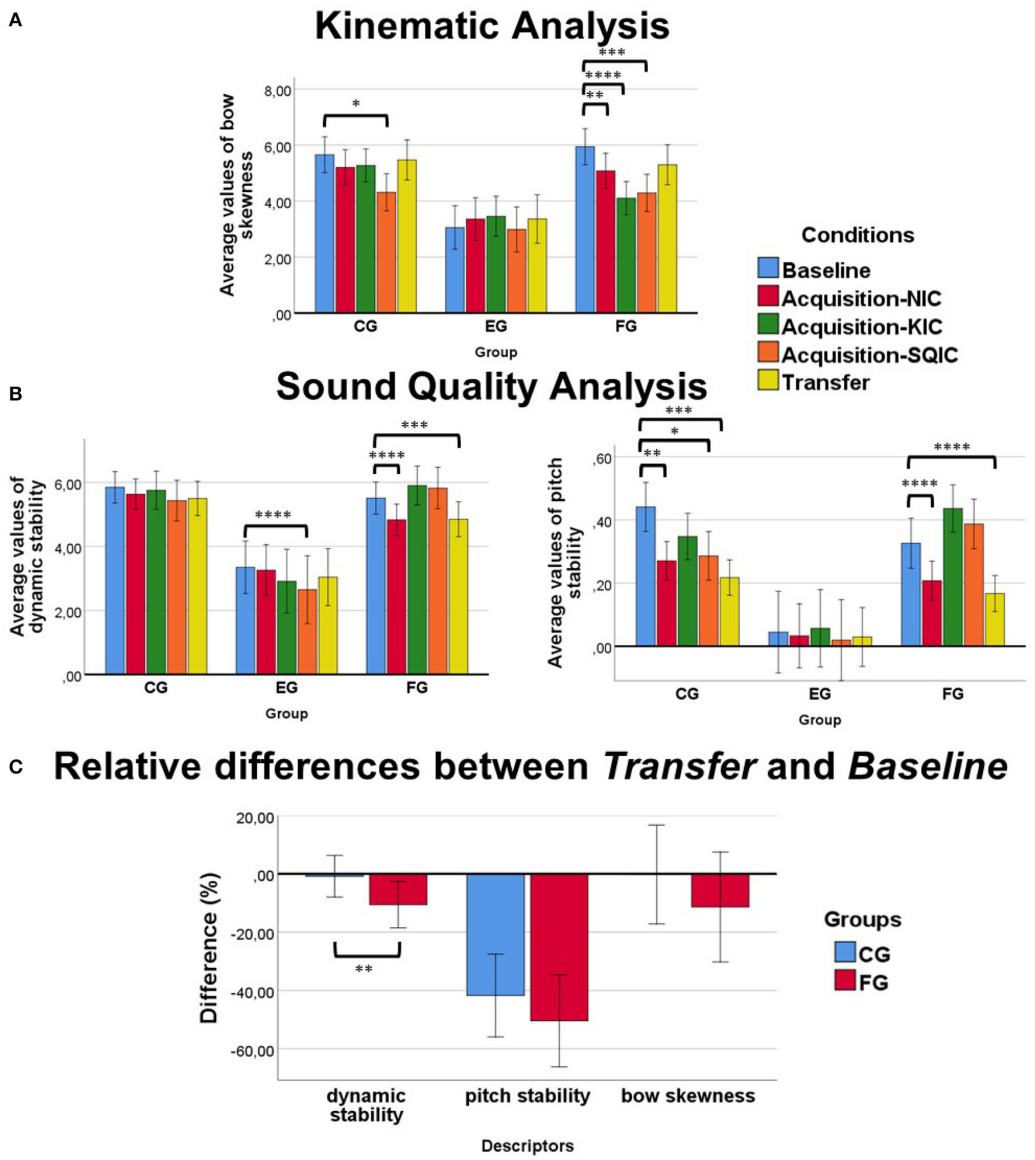
**(A)** Kinematic analysis. *Bow skewness* results: the FG improved significantly their results compared with the *Baseline* at the moment of receiving online feedback on bow motion (i.e., at the *Acquisition-KIC*) and in the rest of the conditions from the *Acquisition* phase. The CG improved their results only at the *Acquisition-SQIC*. **(B)** Sound Quality Analysis. *dynamic stability* results (left): although results for the FG tended to get worse at the moment of receiving online feedback, those results were transferred to conditions without feedback (*Acquisition-NIC* and *Transfer*). No significant improvements were found for the CG. *pitch stability* results (right): both groups of beginners (control and feedback) improved their results in *pitch stability* at the *Acquisition-NIC* and *Transfer*. The FG tended to get worse results when receiving online feedback. **(C)** Relative differences between *Baseline* and *Transfer*: The FG seemed to improve, on average, more than the CG in all the descriptors. However, only significant results between groups were found at *dynamic stability*. *p ≤ 0.05, **p ≤ 0.01, ***p ≤ 0.001, ****p ≤ 0.0001.

Multivariate tests of within-subject effects for *dynamic stability* and *pitch stability* showed significant results in Conditions (*p* < 0.0001) and interaction between Conditions*Group (*p* < 0.0001). Results for *dynamic stability* and *pitch stability* were lower for the EG compared with both FG and CG regardless of conditions (see Figure 5B). That is, the sound of the experts was more stable during the exercise. Tests of Between-Subjects Effects showed significant results for Group both at *dynamic stability* and *pitch stability* (*p* < 0.0001 in both). *Post-hoc* tests showed significant differences between the EG and both the CG and FG in the two descriptors (*p* < 0.0001 in all the tests). Thus, we also considered *pitch stability* and *dynamic stability* as good evaluators of performance and proceeded with the analysis.

Univariate tests of within-subject effects for *bow skewness* showed significant results for Condition (*p* < 0.0001) and interaction between Condition*Group (*p* < 0.0001). Results for *bow skewness* were lower for the EG compared with both FG and CG regardless of conditions (see Figure 5A). Tests of between-subjects effects showed significant results for Group (*p* < 0.0001). That is, their bow was straighter during the exercise. *Post-hoc* tests showed significant results between the EG and the FG (*p* = 0.001) and CG (*p* < 0.0001). Thus, we considered *bow skewness* as a good evaluator of performance and proceeded with the analysis.

### 4.2. Kinematic Analysis

Univariate tests of within-subject effects for the beginner's groups showed significant results for Condition (*p* < 0.0001) and an interaction Condition*Group (*p* < 0.007). *Post-hoc* tests did not show significant differences between CG and FG. Simple main effect analysis revealed significant results for Condition in both the univariate tests of within-subject effects for the FG and CG (*p* < 0.0001 and *p* = 0.003, respectively). Both groups improved on average their results after the *Baseline* (see Figure 5A). The biggest improvements for the FG were found at the *Acquisition-NIC* (14.5% of improvement over the *Baseline*), *Acquisition-KIC* (31% of improvement), and at the *Acquisition-SQIC* (27.8% of improvement). The biggest improvements for the CG were found at the *Acquisition-SQIC* (23% of improvement). Pairwise comparison tests between conditions revealed significant differences between the *Baseline* and the *Acquisition-NIC*, the *Acquisition-KIC* and the *Acquisition-SQIC* in the FG (*p* = 0.002, *p* < 0.0001 and *p* = 0.001, respectively), while in the CG we only found differences between the *Baseline* and the *Acquisition-SQIC* (*p* = 0.019).

### 4.3. Sound Quality Analysis

Multivariate tests of within-subject effects showed significant results for Condition (*p* < 0.0001 in both descriptors) and an interaction Condition*Group (*p* = 0.002 for *dynamic stability* and *p* < 0.0001 for *pitch stability*). *Post-hoc* tests did not show significant differences between CG and FG. Simple main effect analysis revealed significant results for Conditions in the univariate tests of within-subject effects in *dynamic stability* and *pitch stability* for the FG (*p* < 0.0001 in all the tests). Significant results were found only for *pitch stability* in the CG (*p* < 0.0001). The CG improved their results in *pitch stability* after the *Baseline* (see Figure 5B, left figure). Pairwise comparison tests revealed significant differences in *pitch stability* between the *Baseline* and the *Acquisition-NIC*, the *Acquisition-SQIC*, and the *Transfer* block in the CG (*p* = 0.005, *p* = 0.015, and *p* = 0.001, respectively). No significant results were found between the *Baseline* and the *Acquisition-KIC*. A similar but less pronounced trend was observed for their results in *dynamic stability* although they did not reach significance. On the other hand, the FG seemed to improve their results in *pitch stability* at the *Acquisition-NIC* and at the *Transfer* condition but worsened its results at both the *Acquisition-KIC* and the *Acquisition-SQIC*, i.e., when receiving RTVF. This trend was similar for *dynamic stability* although less pronounced (see Figure 5B, right figure). Significant results in the FG for *pitch stability* were only found between the *Baseline*, the *Acquisition-NIC*, and the *Transfer* condition (*p* < 0.0001 in both conditions). Additionally, the FG showed significant differences in *dynamic stability* between the *Baseline* and the *Acquisition-NIC* and the *Transfer* conditions (*p* < 0.0001 and *p* = 0.005, respectively).

Interestingly, simple main effect analysis also revealed significant results for Conditions in the univariate tests of within-subject effects in *dynamic stability*. The EG also seemed to improve their results in *dynamic stability* after the *Baseline* but especially in the *Acquisition-SQIC*. Pairwise comparisons showed that the EG showed significant results between the *Baseline* and the *Acquisition-SQIC* condition (*p* < 0.0001) and close to significance between the *Baseline* and the *Transfer* condition (*p* = 0.07).

### 4.4. Effect of Training on Performance Improvement and Correlations

The FG obtained on average better results than the CG when comparing the *Transfer* with the *Baseline* condition (see Figure 5C). In *bow skewness*, the FG improved 5.5% more. In *dynamic stability*, they improved 10.3% more and in *pitch stability* a 8.1% more. However, only significant results between groups of participants were found for the *dynamic stability* descriptor (*p* = 0.003).

No significant correlations were found between the average value of *bow skewness* variable for each participant at the *Baseline* and *Transfer* phase with any of the two different sound descriptors used to evaluate the sound quality (two-tailed Pearson's correlation).

### 4.5. Questionnaires

In this section, we offer different results for the four different parts of the questionnaire participants answered. Two participants from the EG were removed from the analysis since they belonged to the project.

#### 4.5.1. Satisfaction

**Figure 6:**
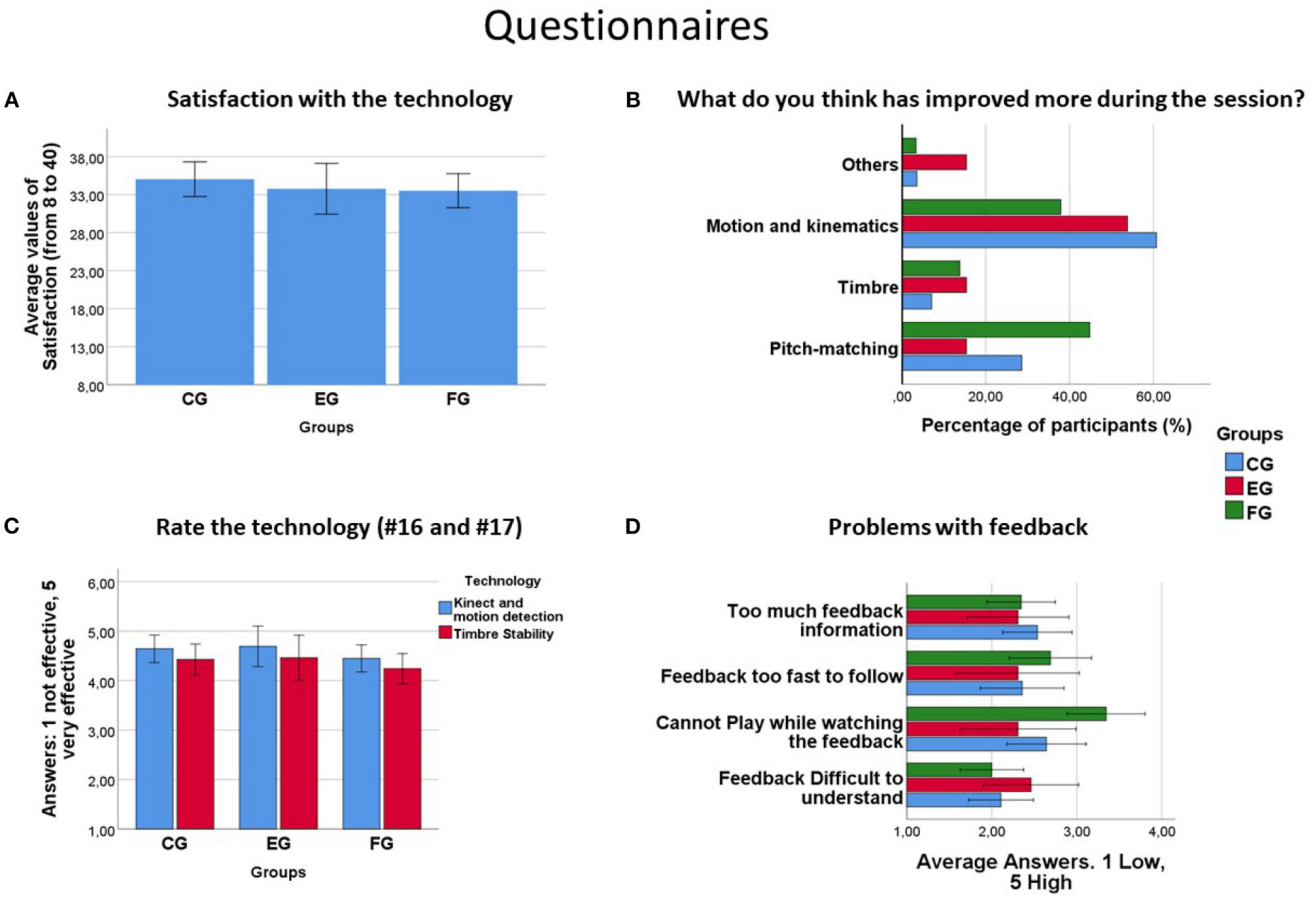
**(A)** Satisfaction with the technology. No significant differences were found and the average values were similar for the three groups. **(B)** Answers regarding the perception of the participant's own performance separated by groups. Just a very small percentage of participants of each group considered that timber stability was the feature that improved more during the session. **(C)** Rate the technology. Effectiveness of each technology according to participants. Both technologies tended to be highly valued by the participants. However, motion capture feedback tended to be slightly more valued. Also, experts tended to rate the effectiveness of each technology better than beginners. **(D)** Problems with feedback. Unlike some participants from the FG and CG, experts did not seem to have problems with feedback. More than half of the participants from the FG especially agreed with the fact that it was hard for them to play while watching the feedback (statement #23).

After adding up the answers of all the participants we got a “Satisfaction Score” which goes from 8 (in case all the answers were 1) to 40 (in case all the answers were 5). A Univariate Analysis of Variance was performed on the data with *Satisfaction* as the dependent variable and *Group* (FG, CG, and EG) as fixed-factor. The average satisfaction with the technology was similar for the three different groups [CG: 34.611 (1.015); FG: 33.517 (1.131); EG: 33.769 (1.689)]. No significant differences were found in the tests of Between-Subjects Effects at *Group*. See Figure 6A.

#### 4.5.2. Participant's Perception of Their Own Performance

In question #18 we asked participants their beliefs regarding what has improved more during the session. Only a small but similar number of participants from the FG and CG (11.1 and 7.7%, respectively) considered that *timber* was the feature that improved more during the session (see Figure 6B). The main differences were found in *motion and kinematics* were a smaller number of participants from the FG compared with the CG (around 17% less) considered it as the most improved feature.

#### 4.5.3. How Effective Is Each Technology

In general, the majority of participants rated both technologies as effective or very effective, even the expert group. Motion capture feedback tended to be more valued than sound quality feedback by all the different groups being the expert group the more optimistic with it. 75% of the experts considered the technology to be “very effective” for learning and 25% of them as “effective” (see Figure 6C).

#### 4.5.4. Problems With Feedback

We found that a relatively constant number of participants (around 20 and 30% from both FG and CG) agreed with the statements “Feedback too fast to follow” and “Too much feedback information” (see Figure 6D). Also, around 10% of participants in both FG and CG agreed with “Feedback difficult to understand.” The expert group tended in almost equal parts to disagree with the statements or to maintain a neutral position.

A clear division is found in the statement “Cannot play while watching the feedback.” More than half of the participants of the FG agreed with that statement vs. 20% of participants of the CG and 0% of the EG.

## 5. Discussion

In this study, we have evaluated the use of RTVF of sound and motion capture technologies by comparing a group of participants practicing with such feedback vs. a group of participants practicing without it. Both groups were composed of beginner violin players. We also asked a group of expert violin players to perform the same tasks for comparison purposes. We replicated some of the results from Blanco and Ramirez (2019) and confirmed the usefulness of the proposed audio descriptors (*dynamic stability* and *pitch stability*) to both differentiate the expert performance from the beginner performance and to track the learning process of participants. Just as *pitch stability* and *dynamic stability* are able to differentiate a beginner from an expert, we have found that differences in *bow skewness* can also differentiate between the two groups.

Regarding the effect of sound quality feedback, both beginner groups improved significantly on *pitch stability* obtaining results close to those of the experts in the *Acquisition-SQIC* condition. No improvement in their results were seen for the expert group nor an effect of the technology in their outcomes. However, the presence of technology seemed to affect the beginner group which used it. Unlike the CG, who learned without RTVF and just focusing on practicing each skill separately, the FG did not show significant improvements in *pitch stability* while using the RTVF technology (neither with kinematic feedback nor with sound quality feedback). This effect may be related to the related distraction that a visual real-time feedback technology can impose in learning the violin, especially with beginners as made explicit by Pardue et al. (2015). Evidence in favor of this hypothesis can be seen in the answers to the questionnaires. More than half of the participants who received RTVF (55.2%) answered “Agree” or “Strongly Agree” in equal proportions to the statement which said “Cannot play while watching the feedback.”

RTVF of sound quality was particularly useful for learning to maintain a stable loudness level through the audio descriptor of *dynamic stability*. Although the CG received the same instructions that the FG about the parameters of the sound that will be considered to evaluate their performance, the CG's results in *dynamic stability* did not improve significantly throughout the session. The FG's results, on the contrary, improved significantly in the *Acquisition-NIC* and in the *Transfer* condition for *dynamic stability*. Again, the fact that the results of *dynamic stability* were worse at the *Acquisition-KIC* and at the *Acquisition-SQIC* may be related to the distracting effect of the RTVF. However, despite the distraction, feedback on *dynamic stability* allowed participants to consider it during their learning as evidenced by their improvement at both the *Acquisition-NIC* and *Transfer* condition. This effect coincides with previous results using RTVF for pitch accuracy in singing voice melody production where the results of performance tend to decay while using the technology but improve at later post-tests scores (Welch et al., 1989; Wilson et al., 2005; Paney and Tharp, 2019). This suggests that, besides the increase of cognitive load that RTVF may impose on participants by worsening their performance while receiving feedback, it should not be considered a damaging factor. As Sherwood and Lee (2003) already pointed out, not only do movements need to be practiced but also the cognitive decision-making processes underlying skilled behavior need practice as well. Despite distracting players' attention, RTVF of sound quality could make explicit performance errors that could be going unnoticed otherwise.

Interestingly, the experts improved their performance significantly in *dynamic stability* while using the RTVF at the *Acquisition-SQIC* suggesting that the technology was not distracting them as much as the beginners. Their ability and their strong formed schemas supposedly would allow them to allocate more cognitive resources to the interpretation of the feedback without disrupting their performance. Again, this was also reflected in the questionnaires where no participant in the EG agreed with the statement “Cannot play while watching the feedback.” Besides, the EG's also seemed to improve more in that descriptor in the rest of the conditions to the point of giving results very close to significance in the *Transfer* condition.

In terms of RTVF of kinematic movements, although participants were told to control three different kinematic variables (*bow skewness, inclination*, and *bow-bridge distance*) for this study we decided to focus only on *bow skewness* which, as already pointed out before, seemed to be a reliable estimator to differentiate a beginner's performance from that of an expert. Both groups of beginners seemed to improve their results after the *Baseline*. The FG improved significantly their results in *bow skewness* at the whole *Acquisition* phase, even in those conditions where the feedback was not present. However, that improvement was not transferred at the *Transfer* condition. On the other hand, results from the CG only improved significantly in the *Acquisition-SQIC*.

Unlike the CG, the RTVF of kinematic movements improved significantly the performance of the FG in the *Acquisition-KIC*. Contrary to previous results with RTVF of sound quality, kinematic feedback seemed to improve the results of participants using it. The reason why performance on *bow skewness* did not decay while receiving feedback was probably due to the nature of the type of feedback itself. The sound quality visual feedback used did not offer information about how to improve the generated sound. On the other hand, kinematic feedback offered real-time information about the movement of the bow allowing participants to immediately correct their bow movements. This distinction between types of feedback is similar to the one we find in visuomotor rotation paradigms between *reward feedback* and *sensory feedback* (Krakauer et al., 2019). Literature in adaptation paradigms reports how each type of feedback could lead to differences in behavior and retention of the learned movements (Izawa and Shadmehr, 2011; Shmuelof et al., 2012; Nikooyan and Ahmed, 2015). This is something that could strongly influence participant behavior and should be taken into account at the moment of designing and evaluating feedback technologies. On the other hand, the fact that performance on *pitch stability* decayed at the *Acquisition-KIC* while *bow skewness* improved in the FG suggests that participants were trying to play with straight bowing at the expense of the quality of the sound. However, it is important to note that for the CG, the performance in terms of *pitch stability* became worse while trying to keep the bow straight but their results in *bow skewness* did not improve significantly as those of the FG.

Both the CG and FG improved their results in *bow skewness* during the *Acquisition-SQIC*. The reasons, however, varied for each group. The CG was able to significantly improve their results both on *bow skewness* and *pitch stability* at the same time. It is possible that by suggesting them to focus only on the quality of the sound, they engaged in an external locus of focus which guided more precisely their arm movements. As Wulf and Lewthwaite (2016) suggested, the external locus of focus prevented learners from interfering with the automatic control processes of their motor system. That could be also the reason why participants from the CG did not improve their results when focusing their attention on their movements. By asking them to focalize their attention on their movements, we would be promoting an internal locus of focus interfering with their automatic control processes. The FG, as mentioned previously, did not improve their results in sound quality during the *Acquisition-SQIC* presumably due to feedback distraction. However, the fact that the FG not only maintained good results at *bow skewness*, but that those results were bigger than for the CG (5% more of improvement) may suggest temporary retention of the kinematic movements needed for straight bowing from the *Acquisition-KIC* to the *Acquisition-SQIC*. The order of the conditions could also have influenced the observed behavior, also in the CG. However, the fact that the FG was able to maintain good results at *bow skewness* during the *Acquisition-NIC* tells us that there was indeed retention at least in the short term that was transferred to the rest of the conditions of the *Acquisition* phase. Moreover, the improvement in *bow skewness* in the *Acquisition-NIC* was accompanied by a significant improvement in both sound quality descriptors. The FG was the only group that showed improvement in all the descriptors at the same time. This suggests that the FG learned how to incorporate together the different feedback received at the *Acquisition-KIC* and at the *Acquisition-SQIC*.

Questionnaires also allowed us to have a broader view of the opinion of participants about the technology. All groups of participants rated both technologies as effective or very effective for learning, especially the EG. In general, motion capture technology tended to be rated as more effective than sound quality feedback. A larger number of participants considered that “Motion and Kinematics” improved more than “Timber” during the session. This contrasts with the obtained outcomes of the experiment where no group retained the levels of straight bowing that they reached at least in one of the three conditions of the *Acquisition* phase. It could be hypothesized that participants from the FG thought that the quality of their sound was not improving because at the time of receiving the feedback they were not receiving a positive one (as inferred from the results in *pitch stability* and *dynamic stability* at the SQIC). At the same time, they improved their bowing movements while using feedback, possibly due to the type of feedback that allowed them to know how to correct their movement. However, the fact that the CG also showed similar results and similar answers in the questionnaires may suggest that straight bowing is a difficult skill to self-assess for those who lack the appropriate metacognitive skills about his/her own level of performance. It also may be unreasonable to expect that learning to bow correctly can be improved in a single session. As Van Der Linden et al. (2011) found, it is even complicated to maintain and retain some of the improvements made during six training sessions. Our results match Linden et al. results by showing how feedback was helping participants to improve their movement. However, although we have seen how this improvement in *bow skewness* came at the cost of disregarding the sound quality of the performance at the moment of receiving feedback, we have also shown how it was retained in conditions where feedback was not present and, accompanied by improvement in sound quality.

SkyNote has been applied at the Royal College of Music with high-level violin students. The results of using SkyNote as well as how the technology can be implemented in teaching and learning practice at a higher education institution will be discussed in an accompanying paper.

## 6. Conclusions

In this work, we have presented and evaluated some of the technologies developed during the TELMI project. We have designed an experimental setup where complete beginners start learning the basics of violin playing, such as the production of a stable and sustained sound. This study extends our previous results (Blanco and Ramirez, 2019) and reaffirms the importance and the impact this kind of technologies may have in the process of learning a musical instrument and evaluating different learning methodologies.

In summary, we can list some of the main findings of this study:

1. We have shown how sound quality and motion-capture descriptors, such as *dynamic stability, pitch stability*, and *bow skewness* may characterize part of the participants' improvement in sound production and bowing technique and may be used to evaluate learning interventions.
2. Although *bow skewness* is usually treated as a precondition for obtaining good sound, the results in this study indicate that, for total beginners, this relation is not straightforward. We have seen how focusing on the quality of the sound rather than focusing on playing with a straight bow could, in fact, improve straight bowing. This fact could be justified by the choice of an external locus of focus (quality of the sound) rather than an internal one (movement of the arm). However, the order of the conditions could have influenced the results.
3. Real-time kinematic feedback of bow movement influenced differently the participant's performance than the sound quality feedback did. While participants improved their bow movements at the moment of receiving kinematic feedback their results in sound quality got worse. Furthermore, their results in sound quality worsened at the moment of receiving sound quality feedback while their bow movements held up better despite not receiving kinematic feedback. However, when RTVF was removed participants improved in all the descriptors. Again, although the order of conditions could have influenced the results we argue that the type of feedback (and modality) is the main reason for these results. Visual feedback splits attention and can lead to an increase in cognitive load in beginners. This is corroborated by the fact that the expert performance was not influenced by real-time feedback. Even more, real-time feedback improved their performance in *dynamic stability* right at the moment it was received.
4. Finally, we have seen how beginners who received feedback tended to improve more, on average than those who did not in the retention test (*Transfer* condition). However, only significant results were found for *dynamic stability* where the improvement was greater and clearer. Interestingly enough, experts also seemed to slightly improve their performance in *dynamic stability* at the *Transfer* condition. However, that improvement was not statistically significant and we cannot directly infer that feedback was the cause.

Such technologies may help students to avoid bad habits that could occur during their long-periods of self-study, and to increase their motivation and own-expectations toward learning. Furthermore, these technologies can be used to better comprehend and add more clarity to the scarce research in motor learning in music activities. Only by improving the ways we can acquire and track data, and extract and evaluate descriptors from activities, which were previously evaluated based on solely subjective mechanisms, we can objectively gain new insights on how the *body*, understood in its entirety, becomes the subject of learning.

## Data Availability Statement

The datasets generated for this study can be found in http://doi.org/10.5281/zenodo.4554201 and in http://doi.org/10.5281/zenodo.4553940.

## Ethics Statement

The studies involving human participants were reviewed and approved by Conservatoires UK Research Ethics committee. The patients/participants provided their written informed consent to participate in this study.

## Author Contributions

AB and RR designed the methodology of the study. AB recorded, processed, and analyzed the kinematics and audio data, and wrote the paper. RR supervised the study and contributed to the writing of the paper. ST supervised the statistical methods used and contributed to the writing of the paper. All authors contributed to the article and approved the submitted version.

## Conflict of Interest

The authors declare that the research was conducted in the absence of any commercial or financial relationships that could be construed as a potential conflict of interest.

## Footnotes

^1^telmi.upf.edu.

^2^https://youtu.be/mUz8fIc1FaY.

^3^https://www.survio.com/survey/d/N2C1Q8P3E9Y4H4A5I.
